# When, how and why BRCA1 and BRCA2 genetic testing is offered to patients who do not meet standard criteria

**DOI:** 10.1186/1897-4287-10-S2-A65

**Published:** 2012-04-12

**Authors:** J Duffy, S Greening, B Creighton

**Affiliations:** 1 Hereditary Cancer Clinic, Prince of Wales Hospital, Australia; 2 Hereditary Cancer Clinic, Wollongong Hospital, Australia; 3 Cancer Care Centre, St George Hospital, Australia; 4 Familial Cancer Service, Royal North Shore Hospital, Australia

## 

Various *BRCA1* and *BRCA2* mutation probability prediction models exist which can be used to determine equitable access to publicly funded genetic testing. In January 2009 two of these models were introduced in three New South Wales genetic services. From this time, the criteria below were used to determine eligibility for publicly funded *BRCA1* and *BRCA2* mutation analysis.

Individual diagnosed with breast or ovarian cancer with one or more of the following:

• Combined Manchester score of 15 or higher

• Combined BOADICEA score of 10% or higher

• Synchronous or metachronous breast and ovarian cancer

• Serous fallopian tube cancer

Cases not fulfilling the above criteria could be offered testing following discussion and approval at a combined monthly meeting of clinical staff. This was instigated because staff thought that some clinical scenarios would not easily fit into the BOADICEA or Manchester models and yet should be considered for testing.

We have audited all 77 cases discussed at the meetings between January 2009 and December 2010. Some cases discussed did in fact meet the testing criteria above. In 41 cases the decision was made to offer publicly funded *BRCA1* and *BRCA2* mutation analysis.

Reasons cases were considered for ‘off-criteria’ testing and bought to the meeting for discussion included:

• Very early age of diagnosis

• Pathological subtype

• Limited family structure

• Non-Western European ancestry

• Affected with prostate or pancreatic cancer

• Ashkenazi Jewish ancestry but no *BRCA1* or *BRCA2* founder mutation identified

We will present an analysis of the 77 cases including case and family history, pathology, BOADICEA score, Manchester score, outcome of discussion, and genetic test results. We will also consider the cost-effectiveness of ‘off-criteria’ testing.

